# Can screening and brief intervention lead to population-level reductions in alcohol-related harm?

**DOI:** 10.1186/1940-0640-7-15

**Published:** 2012-08-28

**Authors:** Nick Heather

**Affiliations:** 1 Emeritus Professor of Alcohol & Other Drug Studies, Northumbria University, Newcastle upon Tyne, NE1 8ST, United Kingdom

## Abstract

A distinction is made between the clinical and public health justifications for screening and brief intervention (SBI) against hazardous and harmful alcohol consumption. Early claims for a public health benefit of SBI derived from research on general medical practitioners’ (GPs’) advice on smoking cessation, but these claims have not been realized, mainly because GPs have not incorporated SBI into their routine practice. A recent modeling exercise estimated that, if all GPs in England screened every patient at their next consultation, 96% of the general population would be screened over 10 years, with 70-79% of excessive drinkers receiving brief interventions (BI); assuming a 10% success rate, this would probably amount to a population-level effect of SBI. Thus, a public health benefit for SBI presupposes widespread screening; but recent government policy in England favors targeted versus universal screening, and in Scotland screening is based on new registrations and clinical presentation. A recent proposal for a national screening program was rejected by the UK National Health Service’s National Screening Committee because 1) there was no good evidence that SBI led to reductions in mortality or morbidity, and 2) a safe, simple, precise, and validated screening test was not available. Even in countries like Sweden and Finland, where expensive national programs to disseminate SBI have been implemented, only a minority of the population has been asked about drinking during health-care visits, and a minority of excessive drinkers has been advised to cut down. Although there has been research on the relationship between treatment for alcohol problems and population-level effects, there has been no such research for SBI, nor have there been experimental investigations of its relationship with population-level measures of alcohol-related harm. These are strongly recommended. In this article, conditions that would allow a population-level effect of SBI to occur are reviewed, including their political acceptability. It is tentatively concluded that widespread dissemination of SBI, without the implementation of alcohol control measures, might have indirect influences on levels of consumption and harm but would be unlikely on its own to result in public health benefits. However, if and when alcohol control measures were introduced, SBI would still have an important role in the battle against alcohol-related harm.

## Introduction

The term “brief intervention” has been applied both to shorter forms of treatment delivered in specialist alcohol or addiction services and to interventions offered by generalist professionals to individuals who are not seeking help for an alcohol problem but whose alcohol consumption is of concern. It has been argued that these two uses of the term should be clearly distinguished [1,2], and it is the second that is the focus of this article. In this sense, it is better to use the term “opportunistic brief intervention,” with the implication that the opportunity is taken of the drinker’s presence in a setting not directly connected with help for alcohol problems to offer advice and assistance to cut down consumption or abstain. For convenience, however, this form of activity will be called simply brief intervention (BI) here.

Brief intervention is directed at two separate targets: hazardous drinkers, whose level or pattern of drinking increases the risk of alcohol-related harm, and harmful drinkers, who have already incurred some degree of harm but who show no or only low levels of alcohol dependence [3]. Since, by definition, these individuals are not seeking help to resolve an alcohol problem, it is necessary to screen for level of consumption and/or the presence of alcohol-related problems or employ some other form of identification before BI can be offered. The more specific aims of screening and brief intervention (SBI) will be discussed below.

There is no single form of BI; rather, the term applies to a family of interventions that differ in length, content, and in a number of other ways [4]. Recently, however, two distinct forms of BI have emerged: brief structured advice and brief motivational interviewing*.* Brief structured advice is typically employed in time-limited situations, such as general medical practice, with immediate intervention following screening. The intervention typically lasts between five and 10 minutes and usually consists of a standard package involving information on drinking risk levels, the status of the patient’s own drinking in relation to those levels, encouragement to cut down and set a date for doing so, and a few simple hints on how cutting down might best be achieved, often accompanied by self-help material. Brief motivational interviewing is a more flexible and client-centred approach that explicitly avoids giving advice unless it is requested. It usually lasts between 20–40 minutes in a single session and often includes follow-up sessions. Based on the principles and techniques of motivational interviewing [5], it was originally described in the early 1990s [6] and has been applied in settings and situations where there is more time for intervention and where staff trained in the approach are available.

Evidence for the effectiveness of SBI is good for primary health care [7], mixed for emergency services and general hospitals wards [8,9], and virtually nonexistent for other medical and nonmedical settings [10]. For purposes of this discussion, however, and based on the assumption that evidence for effectiveness in primary health care can represent effectiveness among hazardous and harmful drinkers in general, BI will be regarded as effective with a number-needed-to-treat (NNT) of 10 [11]. It is also arguable that a public health benefit of SBI, if it is possible, would come about mainly through implementation in primary health care, because that would impact upon the majority of individuals in the population.

### Aims

The aims of this article are as follows:

1) to trace the history of claims for the public health benefits of SBI for hazardous and harmful alcohol consumption (hereafter called excessive drinking).

2) to consider the conditions under which a population-level benefit of SBI could arise.

3) to examine evidence on recent attempts to achieve widespread implementation of SBI, the consequences of those attempts, and the difficulties they have encountered.

4) to contribute to the fourth objective of the International Network on Brief Interventions for Alcohol & Other Drugs (INEBRIA); that is,”To promote the integration of the study of brief interventions with the wider context of measures to prevent and reduce alcohol-related harm.”

5) specifically, to consider the relationship between SBI and alcohol control measures.

6) overall, to ask whether implementation of SBI can lead to population-level reductions in alcohol-related harm, to consider how this question could be answered, and to offer a tentative answer to it.

Before proceeding, two general points should be made. First, classic definitions of public health (e.g., "the science and art of preventing disease, prolonging life, and promoting health through the organized efforts of society" [12]) can encompass a variety of activities and outcomes. For the purposes of this article, the term “public health benefit” refers to the favorable effects of policies and interventions that are detectable in population-level measures of, in this case, alcohol-related harm. Second, although there is a great deal of high-quality information from the US, Canada, and other parts of the world that is relevant to the aims of this article, we will be mainly concerned here with what the author knows best—evidence and policies from European countries and, in particular, from the UK.

### Clinical versus public health justifications for alcohol SBI

There are two ways of justifying the range of activities that come under the heading of alcohol SBI. The clinical justification is concerned with the interests of the individual patient or client who receives the BI. The clinical benefits are in terms of early intervention before harm or, in the case of existing harmful drinking, more severe harm has occurred and secondary prevention before more intensive intervention may be required. Brief intervention can also reduce the current level of alcohol-related harm an individual may be experiencing. An NNT of 10 compares very favorably with general practitioner’s advice to quit smoking [13]. Even if the patient does not respond at first, the intervention may start a process described by movement along the stages of change in the Transtheoretical Model [14]. This kind of justification is of greatest appeal to clinicians, therapists, and counselors concerned with the health and welfare of the patients or clients they encounter in their practices.

The public health justification is not concerned with the interests of individuals but with those of societies and, more specifically, with reducing the aggregate of alcohol-related harm in the population at large. This is achieved by reducing levels of alcohol-related problems *per se* and also by decreasing levels of alcohol consumption in the population linked to a wide range of physical and mental ill-health [15]. It is reckoned that the widespread delivery of SBI among excessive drinkers would be a comparatively cost-effective way of reaching these goals [16]. Although clinicians may recognize these wider benefits of delivering SBI, they are mainly of interest to public health specialists and policy makers.

An objection at this point might be that the distinction above is artificial because, if enough individuals respond positively to SBI, this would amount to an aggregate public health benefit. This is true in a trivial sense. However, the premise here is that a form of intervention applied to individuals can only be described as having a public health benefit if its effects are clearly detectable in population-level measures of harm—in this case, for example, in rates of alcohol-attributable mortality or morbidity, rates of drunkenness or drunk-driving offenses, or measures of alcohol consumption from random surveys of the population or relevant community samples.

If the distinction is accepted, an important point to note is that the clinical justification is independent of the public health justification. In other words, if it were shown that widespread SBI had no detectable public health effect at the population level, this would in no way undermine the clinical justification for offering SBI to individual patients or clients with the aim of improving their health, welfare, and quality of life.

### Early claims for the public health potential of SBI

The trial of physician advice to quit smoking by Michael Russell and colleagues reported in the *British Medical Journal* in 1979 [17] is rightly regarded as a landmark study of BI for substance use disorders and for the new public health more generally. It was one of the main influences on the development and trial in Scotland in the early 1980s of the first alcohol BI pack for use in primary health care (the DRAMS pack [18]). (The European origins of alcohol BI have been recently described by Heather [19].) Russell and colleagues randomly allocated 2138 smokers attending one of 28 general practice surgeries in London to one of four conditions: nonassessment controls; questionnaire-only controls; advised by the general practitioner (GP) to stop smoking; and advice, receipt of a leaflet, and warning of follow-up. The quit rates one year after intervention for these four groups were, respectively: 0.3%, 1.6%, 3.3%, 5.1%. The authors summarized the implications of their findings as follows: “The results suggest that any GP who adopts this simple routine [i.e., the fourth group] could expect about 25 long-term successes yearly. If all GPs in the UK participated, the yield would exceed half a million ex-smokers a year. This target could not be matched by increasing the present 50 or so special withdrawal clinics to 10,000” (p.231).

This is probably the earliest statement of the public health potential of SBI for reducing the harm from any kind of unhealthy behavior. However, it is clear that, had the authors’ expectations been realized, the smoking habit in the UK would have been entirely eliminated some years ago! (It would have resulted in 16 million ex-smokers in the 32 years since the *BMJ* article; the current prevalence of smoking in the UK is approximately 10 million.) Reasons why this has not happened are not hard to find. First, there are the well-known difficulties in extrapolating the results of randomized controlled trials to the real world of everyday practice. Second, although smoking prevalence has declined in many industrialized countries, experience has revealed a recalcitrant rump of smokers who are harder to shift from their habit [20]. Third, and most obviously, not all GPs in the UK have adopted the “simple routine” in question, or anything like it.

For the purposes of this article, a UK smoking research expert, Dr. Martin Jarvis, was asked for his assessment of the contribution BI has made to reductions in smoking prevalence over the last 20 to 30 years. He replied that the main driver of reduced prevalence has been a change in the social climate surrounding smoking; i.e., the “denormalization” of smoking. This had mainly been brought about by advertising bans and smoke-free legislation, but price increases have also been important. Against this background, it is difficult to disentangle the effects of GPs’ advice and treatment in general, but uptake of BI by smokers is far lower than it should be, and GPs and other health professionals do not offer BI as often as they should. The extent to which these remarks transfer to desired reductions in the prevalence of excessive drinking will be returned to.

The first well-resourced trial of GP-delivered SBI, resulting in the first good evidence for its effectiveness in any setting, was carried out by Paul Wallace and colleagues using the Medical Research Council GP Research Network [21]. They randomly allocated 909 heavy drinking patients from 47 group practices throughout the UK to one of two groups: 1) advice and information about reducing consumption plus a leaflet and up to five additional sessions at the discretion of the GP; or 2) a nonintervention control. At one year follow-up, the proportion with excessive consumption had dropped by 43.7% in the intervention group compared with 25.5% in controls, a difference that was highly significant. In what was no doubt a reflection of the earlier summary of the Russell et al. study [17], Wallace and colleagues concluded that, “If the results of this study were applied to the UK, intervention by GPs could each year reduce to moderate levels the alcohol consumption of some 250,000 men and 67,500 women who currently drink to excess. GPs and other members of the primary health care team should therefore be encouraged to include counseling about alcohol consumption in their preventive activities” (p.663).

Again, if this potential had been realized, it would have resulted in 5.75 million heavy drinking men and 1.55 million heavy drinking women becoming low-risk drinkers since Wallace et al.’s 1988 publication. Needless to say, this has not happened. The reasons are similar to those applying to smoking cessation (see above) but chiefly because most GPs in the UK have not incorporated alcohol SBI into their routine work.

### Modeling SBI effects

A more recent and sophisticated attempt to predict the effect that widespread implementation of alcohol SBI in primary health care would have on rates of excessive drinking in the UK was made by a group from the School of Health and Related Research at the University of Sheffield [22]. This work was commissioned by the National Institute for Health and Clinical Excellence (NICE) in conjunction with the development of public health guidance to promote the prevention and early identification of alcohol use disorders in adults and adolescents [23,24].

The Sheffield model involved integrating routine registration and attendance data in general practices and emergency departments, cost information, linking scores on the Alcohol Use Disorders Identification Test (AUDIT) [25] with baseline consumption levels, and published research evidence on the effectiveness of BIs, principally the Cochrane meta-analysis by Kaner and colleagues [7]. Estimates were made of the effects of a set of possible policies implemented over an assumed 10-year national screening program in England by quantifying the costs of implementation, the effects on 47 health conditions, and savings in health care costs. Crime and workplace harms were excluded. This project was primarily an exercise in economic modeling, and, although cost-effectiveness data are of interest, we will be mainly concerned here with the modelling of SBI implementation and its effects.

All screening was considered to be opportunistic, and an arrival profile was estimated for the proportion of each population subgroup that would attend in the first year of the screening program; repeat screening at subsequent attendances was assumed not to occur. Screening was assumed to be carried out using the full AUDIT [25], the AUDIT-C (the three AUDIT consumption questions) [26], or the Fast Alcohol Screening Test (FAST) [27]. Cost-effectiveness ratios were estimated in terms of health care costs per quality-adjusted life year (QALY) gained, similar to a standard NICE technology appraisal. Three general scenarios for SBI were examined: 1) at the next GP registration (i.e., when patients change their GP); 2) at the next GP consultation; and 3) at the next consultation in an emergency care setting. Table 1 shows the main assumptions underlying each scenario and their estimated effects over a 10-year period.

**Table 1:** Three intervention models from the Sheffield Alcohol Policy Model, version 2

	**Next GP Registration**	**Next GP Consultation**	**Next ED Consultation**
**Baseline Scenarios**	Practice nurse undertakes both screening and, where appropriate, BI	Physician undertakes both screening and, where indicated, BI as part of consultation	Prescreen applied depending on reason for attendance or diagnosis, screening prior to discharge, BI offered as a separate appointment on day after screening
	► Screening using full AUDIT followed by 25-minute intervention	► Screening using full AUDIT followed by 25-minute intervention	► Assume 30% take-up of BI, prescreen similar to PAT, screening with FAST, followed by 50-minute intervention
	► Screening using AUDIT-C followed by 5-minute intervention (similar to DES configuration)	► Screening using AUDIT-C followed by 5-minute intervention (similar to DES configuration)	
	► Screening using FAST followed by 5-minute intervention	► Screening using FAST followed by 5-minute intervention	
**Results**	In all three cases, estimated costs of delivering BI outweighed by financial savings due to subsequent reduced burden of illness	For 25-minute BI, estimated costs outweigh health-care costs avoided, with net cost overall and ICER of £5,900 per QALY gained (i.e., cost-effective)	ICER estimated at £9,700 per QALY (i.e., cost-effective)
	QALY gains and, therefore, baseline interventions estimated to be better than “doing nothing”	For 5-minute BI, intervention costs lower and ICER improved, i.e., cost-savings	Despite 10-year program involving screening over three-fourths of adult population, only 18% of hazardous/harmful drinkers estimated to receive BI due to low take-up rate of 30%
	Screening on next registration estimated to apply to 39% of population of England over 10-year period, with one-third of hazardous and harmful drinkers being screened, detected, and given BI	Different from next GP registration because	
		► GP staff costs higher than those of practice nurse	
		► Men consult less frequently than women	
		► Patients consult GP much more frequently than they change GP	
		Thus, 96% of population screened over 10 years (the majority in the first year) with 70-79% hazardous/harmful drinkers receiving BI	
		Estimated gain is over 100,000 QALYs over 10-year screening program	

GP = general practice; ED = emergency department; BI = brief intervention; AUDIT = Alcohol Use Disorders Identification Test (AUDIT-C = three AUDIT consumption questions); DES = direct enhanced service; PAT = Paddington Alcohol Test; FAST = Fast Alcohol Screening Test; ICER = incremental cost-effectiveness ratio; QALY = quality-adjusted life year.

Despite the cost-effectiveness of the emergency department scenario shown in Table 1—i.e., an incremental cost-effectiveness ratio (ICER) of £9,700 per QALY—being within standard NICE guidelines, the results for this scenario are disappointing. While it was calculated that 78% of the adult population would be screened in this setting within 10 years, because of an estimated take-up rate of just 30%, only 18% of excessive drinkers would receive a BI. It is also relevant that evidence for effectiveness of SBI in emergency care is inconclusive [8]. Consequently, we focus here on the two GP-based scenarios that were applied to the English population aged ≥11.

There is evidence of the cost-effectiveness of SBI in both GP scenarios in Table 1, with cost-offsets for the next GP registration (i.e., estimated costs of delivering SBI were outweighed by financial savings due to a subsequently reduced burden of illness). The main comparison of interest between these two scenarios for present purposes, however, concerns the proportions of the general population who would be screened and the proportion of excessive drinkers in the population who would receive a BI. Thus, screening at the next registration was estimated to apply to 39% of population of England over a 10-year period, with one-third of hazardous and harmful drinkers being screened, detected, and given a BI. In the Next GP consultation scenario, it was estimated that 96% of the population would be screened over 10 years (the majority in the first year), with 70-79% of hazardous/harmful drinkers receiving a BI.

In considering the findings of this economic model, the many assumptions underlying it should be borne in mind. For example, it was assumed that all patients visiting their GP over a 10-year period would be screened (but only once), all those screening positive would be offered a BI (but only once), and all those offered a BI would accept it. The assumptions that screening and the offer of BI would occur only once in routine practice might be considered unrealistic. The further assumption that mean alcohol consumption in the years following a BI can be adjusted by using a “rebound to baseline” of seven years, based on data reported in the US by Fleming and colleagues [28], may be regarded as especially questionable in the UK context. The impacts on morbidity and mortality were estimated by a “consumption-to-harms” model, but the relationship between consumption and screening score was based on limited survey data. While the effects of BI of different lengths were estimated from the Kaner et al. meta-analysis [7], the effects of possible booster sessions were excluded, and there were assumed to be no variations in BI effectiveness when delivered by different staff members. If incorrect to various degrees, some of these assumptions would result in overestimation of the cost-effectiveness of SBI and some in underestimation.

Despite the qualifications and misgivings that arise from the Sheffield economic model, its findings are useful for suggesting two broad conclusions. First, because of its limited reach, SBI confined to new patient GP registrations is likely to result in only a modest reduction in the prevalence of excessive drinkers in the population, and, assuming a BI success rate of 10% [3], one that may not be detectable on population-level measures. On the other hand, because of its much wider penetration, opportunistic screening at each patient appointment would be likely to have a detectable impact on the prevalence of hazardous and harmful drinkers in the population and would be a cost-effective means of achieving this impact.

### Public health benefit presupposes widespread screening

#### Targeted versus universal screening

If a population-level benefit of SBI is possible, the findings of the Sheffield model suggest that it must be based on widespread screening, including nearly the whole population and, thus, the majority of excessive drinkers. However, recent English government policy favors *targeted* over *universal* screening in general practice and other medical settings. In a Direct Enhanced Service [DES] commissioned through the Primary Care GMS Contract, GPs are directed to offer an alcohol screen to all new registrants aged 16 and older [29]. (There are a few local variations to this; e.g., NHS South of Tyne & Wear has introduced a Local Enhanced Service that gives payment for universal screening but also gives guidance on targeted groups.) In this way, the DES for alcohol SBI compares unfavorably with the set-up for smoking cessation interventions that are included as part of the Quality and Outcomes Framework (QOF), in which GPs are rewarded for intervening among all smokers in the practice.

In Scotland, where a more severe national alcohol problem is reflected in more government action compared with the other UK countries, there has been a health service target of delivering 149,449 BIs during the period 2008/09–2010/11 [30]. This target has been met (174,205 interventions delivered) and has been extended for another year. This is commendable, but the target is based on clinical presentation and new registrations, not on universal screening.

Guidance issued by NICE recommends that NHS professionals should carry out universal screening if possible; but if not, they should conduct targeted screening in clinics and presentations where heavy drinking patients are most likely to be found [23]. The recourse to targeted screening by NICE and in national policies is partly a reflection of evidence that health care professionals much prefer targeted screening and often find universal screening unacceptable [31]. It must also be said that there is considerable resistance to universal screening from some sections of the medical profession, presumably due to the increased workload it implies. This tendency was encouraged by the 2003 publication in the *BMJ* of an article by Beich and colleagues from Denmark [32] alleging that screening in general practice “does not seem to be an effective precursor to brief interventions targeting excessive alcohol use” (p. 536). This conclusion is debatable, but the main point is that the public health potential of SBI is unlikely to be realized without the widespread deployment of universal screening.

#### A nationwide screening program for excessive drinking?

The concept of universal screening, wherein individual medical practices and practitioners are reimbursed or otherwise take responsibility for delivering SBI, is different from that of formal nationwide screening program. In the UK, a national screening program represents a systematic attempt by central government, carried out mainly via general medical practice, to screen the entire population, or the relevant parts of it, for early signs of specific conditions. Proposals for such a program are assessed by the UK National Screening Committee (NSC) using a standard set of 13 criteria that any condition must meet to be accepted for mass screening. Conditions that have passed this test and are now targets for mass screening include breast and/or cervical cancer among women, congenital heart disease among the newborn, and bowel cancer among all adults. An appraisal of the suitability of “alcohol misuse” against these criteria was recently carried out by the organization Solutions for Health on behalf of the Department of Health [33]. Alcohol misuse was not recommended for a formal NSC screening program, and it is relevant here to ask why.

The criteria used by the NSC are shown in Table 2 together with a “verdict” for each from the appraisal. The appraisal is a curious document in some ways because, for the majority of criteria, it is not explicitly stated whether or not the criterion in question has been met or has failed. While some of the criteria appear from the text to be met by alcohol misuse and others appear questionable, yet others that seem unlikely to be met are not given as a reason for rejection; e.g., “All the cost-effective primary prevention interventions should have been implemented as far as practicable” [4].

**Table 2:** Criteria used by the UK National Screening Committee for the appraisal of a new formal screening program and verdict applied to alcohol misuse

**Criterion**		**Verdict**
1. The condition should be an important health problem		√
2. The epidemiology and natural history of the condition, including development from latent to declared disease, should be adequately understood, and there should be a detectable risk factor, disease marker, latent period, or early symptomatic stage		?
3. If the carriers of a mutation are identified as a result of screening, the natural history of people with this status should be understood, including the psychological implications		N/A
4. All the cost-effective primary prevention interventions should have been implemented as far as practicable		?
5. There should be a simple, safe, precise, and validated screening test		X
6. The distribution of the test values in the target population should be known and a suitable cut-off level defined and agreed		X
7. The test should be acceptable to the population		?
8. There should be an agreed policy on the further diagnostic investigation of individuals with a positive test result and on the choices available to those individuals		√
9. If the test is for mutations, the criteria used to select the subset of mutations to be covered by screening, if all possible mutations are not being tested, should be clearly set out		N/A
10. There should be an effective treatment or intervention for patients identified through early detection, with evidence of early treatment leading to better outcomes than late treatment		√
11. There should be agreed evidence-based policies covering which individuals should be offered treatment and the appropriate treatment to be offered		√
12. Clinical management of the condition prior to participation in a screening program and patient outcomes should be optimized in all health care providers		?
13. There should be evidence from high-quality randomized controlled trials that the screening program is effective at reducing mortality or morbidity		X

√ = criterion met.

X = criterion failed.

? = verdict uncertain.

N/A = criterion not applicable.

The two criteria explicitly identified as grounds for rejection are as follows:

· *Number 5: There should be a simple, safe, precise and validated screening test.* It is concluded that, while the AUDIT [25] is the most validated screening tool, and there is evidence that it is effective in identifying Caucasian men who misuse alcohol, there is no single questionnaire or test that has been validated for all subgroups of the population. Cut-off points have yet to be defined for some subgroups, such as young people, women, cultural minorities, and those over 65. A consequence of using self-reported behavior and behavioral change is that there is no independent measure, such as a biomarker, that can provide a single gold standard against which the performance of screening questionnaires can be measured. This is a prerequisite of a formal screening program set out in the criteria laid down by the NSC. (This basis for rejection seems also to apply to criterion 6 in Table 2.)

· Number 13. *There should be evidence from high-quality randomized controlled trials that the screening program is effective in reducing mortality and morbidity.* The appraisal concludes that there is currently limited evidence and no randomized controlled trials showing that the short-/medium-term reductions in alcohol intake shown in Caucasian men have an impact on morbidity and mortality rates and social harm. A demonstrable reduction in morbidity and/or mortality rates as a result of screening is a prerequisite for a formal screening program.

Any disappointment that might be felt at this decision is perhaps tempered by the fact that other serious health conditions, such as depression and Alzheimer's disease, have also failed to meet NSC criteria for nationwide screening. The direct implications for the alcohol field are that, if it is desired to aim for population-level decreases in alcohol-related harm by means of screening the whole population, screening tests must be developed that meet NSC requirements, and RCTs with sufficient follow-up to demonstrate positive effects of SBI on rates of morbidity and mortality must be published.

### Screening results from the Swedish Risk Drinking Project

While governments around the world have, so far, been reluctant to attempt widespread implementation of SBI throughout national health systems, there are some exceptions. One of these is the Risk Drinking Project carried out in Sweden from 2004 to 2010. This was probably the largest coordinated attempt so far to implement SBI in a single country [34]. The project was based on a government initiative to give alcohol issues a more prominent place in routine primary, maternity, and occupational health care, involving both physicians and nurses. It had total funding of approximately 25 million Euros. Using occupational health care alone as an illustration, 22 courses were carried out teaching the Risk Drinking Model: i.e., screening with AUDIT and a biological marker (carbohydrate deficient transferrin [CDT]), followed by feedback and BI. In total, 530 workers within occupational health care participated in the training. In addition, more than 900 workers participated in half- and whole-day information seminars and network meetings for knowledge and experience exchange within occupational health care. Fourteen national conferences were held across the country in cooperation with the Confederation of Swedish Enterprise, the Swedish Association of Local Authorities and Regions, the employers’ association known as Alecta, and the insurance firm, AFA. In addition, 160 local and regional workplace seminars were held in cooperation with occupational health care units and workplaces nationwide, with approximately 6000 participants. This gives some idea of the ambition and reach of the project.

It should be noted here that occupational health care in Sweden, as well as in Finland and some other European countries, is quite different from the employee assistance programs found in the US. The Swedish working population mainly uses occupational health care as a first point of contact for the treatment of acute and chronic illnesses. Primary health care outside the employment setting is mainly used by children, the elderly, and the unemployed. In this way, occupational health care in Sweden is an integral part of the primary care system.

The Risk Drinking Project was carefully evaluated, and findings are currently being published [35]. In one evaluation study [36], a telephone-administered questionnaire was completed between 2006 and 2009 by a representative sample of approximately 72,000 adults from the Swedish-speaking general population. An “alcohol enquiry” was defined as having been asked about one’s drinking by a physician in any health care visit in the last 12 months. Among “hazardous drinkers” (i.e., all those drinking over recommended limits), 17% of men and 13% of women who had visited a physician in the last year recalled having received an alcohol enquiry, while among “sensible drinkers” (i.e., all those drinking at or below recommended limits), 15% of men and 10% of women did so. Overall, 14% of the sample recalled having received an alcohol enquiry. Although it is encouraging that more hazardous drinkers remembered an alcohol enquiry than sensible drinkers, the fact is that, despite the massive effort to persuade medical practitioners to screen for alcohol consumption, only 14% of the people surveyed could recall being asked about their drinking.

### Public health benefit presupposes widespread implementation of brief interventions

As well as screening, the possibility of a public health benefit from SBI must presuppose the widespread delivery of BI. Recent research from Sweden and Finland, probably the two countries in which SBI has been implemented most widely, is relevant to the issue of the consequences of widespread BI.

#### Brief intervention results from the Swedish Risk Drinking Project

In another evaluation of the effects of the Risk Drinking Project [37], a questionnaire was mailed to a randomly selected representative sample of the Swedish population. The questionnaire asked whether alcohol had been discussed at a health care visit during the past year and, if so, what were the duration, contents, experience, and effects of any conversation about drinking. Of the 66% of the sample who had visited health-care services in the last year, 20% reported having had one or more conversations about alcohol during these visits; this figure was extrapolated to 13% of the Swedish population aged 18–64 years. The duration of conversations was generally brief, and the contents, experiences, and effects varied according to the type of drinker concerned. Reduced alcohol consumption as a consequence of intervention was reported by 12% and, interestingly, was more likely to be reported when the conversation lasted for 1–10 minutes (versus one minute) and included advice on how to cut down. There are encouraging aspects of these results, but, once more, the basic finding is that, despite the enormous effort to implement BI in the Swedish health-care system, only 13% of the population recalled having discussed drinking with a health-care practitioner during the past year.

#### Brief interventions: the Finnish experience

In Finland, there has been a concerted attempt since the early 1990s to increase health professionals’ SBI activity and induce favorable attitudes toward it, accompanied by extensive training and support from researchers. This was supplemented by a government-funded project starting in 2004 known as the National Brief Intervention Project (VAMP) [38]. With funding of 2.5 million Euros, about 2000 nurses and 1000 general medical practitioners were trained and supported to carry out SBI. The participating municipalities represented about 25% of the Finnish population, but there was also a concerted attempt to cover the whole country. There was also a separate Occupational Health Care Project in which an additional 2000 health professionals were trained [38].

A study was mounted to examine how far these widespread and persistent efforts to implement SBI had resulted in the “institutionalization” of SBI activity among Finnish physicians between 2002 to 2007 [39]. In that time, the proportion carrying out SBI increased from 59% to 79%, with regular activity increasing from 9% to 17% and occasional activity from 50% to 61%. Of those who were conducting SBI in 2007, 52% reported increased activity compared with five years earlier. The authors concluded that, despite the continuing challenge of training and motivating those with low SBI activity, implementation among Finnish physicians was reasonably high with an increasing trend.

Another project aimed to evaluate how far this effort had impacted the Finnish general public and how well it reflected public opinion [40]. Face-to-face interviews were carried out in 2008 with a random sample of 2725 Finns aged 15–69 from the general population. The great majority of the sample (over 90%) had positive attitudes toward being asked about drinking, but, of those who had been in contact with health care in the last year, only one-third recalled being asked, and these were predominantly young male heavy drinkers and those of higher socioeconomic status. Of those who had been asked, although 37% overall had been given advice about drinking, 50% of heavy drinkers had received no advice.

In interpreting these findings, the self-report nature of the data should be taken into account. It is possible that respondents either forgot their physicians asked them about alcohol or that respondents did not wish to admit that they had discussed their drinking with their physician due to the stigma associated with alcohol problems. This would suggest that the estimates provided are lower than the real extent of penetration. Nevertheless, this does not alter the conclusion that, despite the substantial effort to institutionalize SBI in the Finnish health care system over the years, and despite positive public opinion regarding alcohol SBI, the extent to which people are being asked about their alcohol consumption, and the extent to which heavy drinkers receive advice about it, is still lower than would be required to produce a population-level reduction of excessive drinking or alcohol-related harm in Finland.

### Research on the relationship between treatment for alcohol problems and population-level harm

Even the most enthusiastic modeller would concede that empirical research is a superior form of evidence. Unfortunately there is a dearth of empirical evidence on the relationship between SBI and population-level measures of alcohol-related harm. There is, however, a small body of research on the relationship between the provision of formal treatment for alcohol problems and population- or community-level harm, and this might be considered relevant to present interests.

The first such study was carried out in Stockholm, Sweden [41], where there had been an impressive decline in inpatient care for liver cirrhosis and pancreatitis from around 1978 to 1984, combined with similar decreases in rates of alcoholic psychosis, alcohol dependence, and alcohol intoxication requiring treatment. Using time-series analysis, the author concluded that most of this decrease could be accounted for by a 20% fall in alcohol sales during the period in question, but that a large increase in the sale and use of disulfiram and calcium carbimide could be implicated too.

A larger scale time-series analysis was carried out by Holder and Parker [42] using data from the US state of North Carolina between 1968 and 1987. Beginning with established evidence that alcohol consumption is an important determinant of levels of liver cirrhosis mortality, the authors enquired why this relationship had not been apparent in some data from the USA and Canada and hypothesized that an increase in treatment for alcohol dependence had resulted in a disjuncture in the relationship between consumption and cirrhosis mortality. This hypothesis was confirmed by the analysis. The authors interpreted their results as showing clearly that treatment for alcohol dependence, provided in sufficient quantity in a large geographical area, can result in significant reductions in liver cirrhosis mortality. This reduction mainly took the form of a short-term lagged effect in which treatment appeared to delay death among individuals with advanced liver disease. Thus, although this is probably the strongest piece of evidence in support of a public health effect of treatment, its relevance to the population of those who typically receive BIs, and who are unlikely to suffer from severe liver disease, must be doubted.

Although the studies above are valuable, this topic is mainly associated with research by Robert Mann, Reginald Smart, and their colleagues from the former Addiction Research Foundation in Toronto, Canada. In their first publication [43], the authors examined changes in treatment for alcohol problems in the Canadian province of Ontario and in per capita alcohol consumption and rates of liver cirrhosis, the latter of which had substantially decreased in the years immediately preceding the research. They concluded that, with the exception of a single area of the province, increases in treatment were linearly related to decreases in hospital discharges for cirrhosis. However, the method of analysis was considered inadequate for the purpose intended by Holder and Parker [42].

In the latest publication on this topic [44], this research group tested the hypothesis that rates of cirrhosis mortality are positively associated with per capita alcohol consumption and negatively associated with rates of membership in Alcoholics Anonymous, again using data from Ontario from 1968 to 1989. Both arms of this hypothesis were supported in the analysis. While rightly warning that the limitations of the study restricted the ability to infer a causal relationship, the authors claim that their findings are consistent with previous research indicating that AA membership can reduce cirrhosis mortality.

Evidence on whether programs for high-risk drinkers, including opportunistic BIs, can have an impact on population or aggregate levels of alcohol problems was reviewed and discussed by Smart and Mann in 2000 [45], together with some useful commentaries on their article. The authors concluded, “Programs for high-risk drinkers can have beneficial aggregate-level effects and are thus a valuable component of population-based efforts to reduce alcohol problems” (p.37). A number of commentaries, however, pointed to the complexity of the relationship in the real world between programs at the individual level, traditional preventive policies, and aggregate measures of harm [46-48]. It should also be noted that these claims for population-level effects concern only alcohol problems *per se*, including 100% alcohol-attributable disease, and not the wider range of mortality and morbidity in which drinking is implicated. The emphasis in this research on rates of liver cirrhosis as a measure of aggregate alcohol-related harm and the limited relevance of this measure to the effects of SBI has already been noted.

Perhaps the most useful conclusion from this body of research for present purposes is that there appears to be no good reason why similar studies could not be carried out on the effects of SBI. If a geographical area can be identified in which the level of SBI activity over time can be accurately recorded, and there exist reliable population measures of alcohol-related harm, preferably beyond cirrhosis mortality rates, then it should be possible to examine the relationship between these two sets of variables and see whether a *prima facie* case can be made for a public health effect of SBI.

### Experimental investigations of population-level effects of SBI

The fundamental limitation of the evidence just summarized is, of course, that it is correlational in nature; thus, it is difficult to adduce causal relationships between variables. Unfortunately, there appear to be no published experimental studies exploring the hypothesis that widespread SBI can lead to population-level effects. In this section, we will briefly consider the kinds of study design that might be directed to this hypothesis.

#### Natural experiments

Researchers should be on the lookout for opportunities to compare aggregate measures of alcohol-related harm from geographical areas and communities in which SBI has been intensively implemented over a protracted period of time with other areas, similar as far as possible on relevant demographic and other background variables, where it has not. This is the weakest form of experimental evidence but better than nothing.

#### Quasi-experimental designs

This involves implementing SBI intensively in a selected area or areas and not implementing it in other areas matched as far as possible on relevant background variables (e.g., sociodemographic composition, baseline level and variability of alcohol consumption etc. [49]). After a stipulated period, identical or at least comparable aggregate measures of harm, including possibly findings from before/after random surveys of the general population, would be compared between experimental and control areas. Interestingly, this kind of design was considered at some length by investigators as the basis for Phase IV of the WHO Collaborative Project on the Identification and Management of Alcohol-related Problems in Primary Health Care [50] before it was concluded that it would be too difficult for a multi-country project of this kind and the action research framework that was eventually used was agreed upon [51]. Nevertheless, a collection of quasi-experiments, designed to discover whether a population-level benefit of widespread SBI could be detected, was thought to be the logical conclusion to a 20-year project in which SBI had been developed and tested.

There are, of course, formidable difficulties with quasi-experimental designs. These include the problem of matching experimental and control areas on possible confounding variables and of controlling the required level of SBI activity (i.e., intensive over time in experimental areas and absent over time in control areas). Nevertheless, quasi-experimental designs have a respectable place in the history of alcohol-problems research and have provided evidence for the effectiveness of community-based prevention projects [52,53]. One study of this type [54] included BI given by GPs as part of a community-based prevention program under study, but it was not possible to isolate the effects of SBI from those of the other program components. However, quasi-experimental designs can, in principle, be applied to the question of public health benefits from SBI and are needed to provide empirical evidence on the question of whether such benefits are large enough to be detected.

#### Cluster randomized controlled trials

This is the most rigorous type of design in this list and again has a respectable place in research on community-based prevention programs [55]. The unit of randomisation here would be communities in which SBI was or was not intensively implemented. While individual patient data would be of interest, the main outcome variables would be aggregate data on alcohol-related harm from each community. Projects of this kind to evaluate the population-level effects of SBI are also needed.

### Necessary conditions for a population-level effect of SBI

Before proceeding to the final section of this article, it will be useful to summarize the conditions that it must be assumed are necessary for a population-level effect of SBI to occur.

1) All or nearly all people in the population are screened, and the great majority of excessive drinkers in the population are identified and offered a BI.

2) BI reduces consumption to low-risk levels in some of those who receive it (around 10% based on present data).

3) Reductions in consumption resulting from BI are relatively long-lasting.

4) There is a dose–response relationship between alcohol consumption and harm such that reductions in consumption among excessive drinkers lead to reductions in harm.

With the exception of the second item, all these necessary conditions are currently unlikely, either because they are difficult to achieve or because there is no evidence to support them. In the case of the first assumption, evidence from Sweden and Finland suggests that, even after thorough, persistent, and expensive national programs to implement SBI, no more than a half of excessive drinkers will receive advice about their drinking.

Even when that advice is received and results in behaviour change, there is little evidence that these changes will be sustained over time. The only good evidence for the durability of low-risk drinking following BI comes from the work of Fleming and colleagues [56] who reported continuing benefits of BI four years after intervention. This study is relied on in nearly every discussion of the long-term benefits of BI in the world literature. In a 10–16 year follow-up of the sample recruited in the well-known Malmö community health screening project, Kristenson and colleagues [57] reported that intervening had reduced mortality among middle-aged men, but it is doubtful whether the original intervention could meaningfully be described as brief. A 10-year follow-up in Australia [58] failed to find evidence that, in the absence of booster sessions, BI could sustain significant long-term reductions in drinking. Thus, much better evidence is needed for the sustained effects of BI before the third condition above can be regarded as met.

In the case of the fourth condition, there is copious evidence that increased consumption is associated with higher rates of mortality, morbidity, and other types of harm. However, this is different from the requirement that reductions in consumption among those who have been drinking heavily, sometimes for many years, are associated with reductions in harm. As discussed earlier, the NSC insisted that more evidence was needed that SBI led to reductions in mortality and morbidity before it could be considered for a mass screening program.

### The political acceptability of widespread SBI

Another necessary condition for a population-level effect might be that widespread implementation of SBI is politically acceptable. Recently in England, the NHS Future Forum, a body set up by the government to provide advice on the future of health services, recommended a transformation in the relationship between NHS staff and patients in order to reduce the toll of ill-health, and the cost to the NHS of rapidly rising numbers of people with ill-health linked to lifestyle [59]. Dubbed “make every contact count,” the proposal is for all health-care professionals—not just physicians and nurses, but surgeons, midwives, health visitors, physiotherapists, pharmacists, dentists, etc.— to routinely talk to patients about lifestyle, even when the presenting problem has no obvious connection with it; and, when required, offer motivationally-based advice on lifestyle change [60]. The aspects of lifestyle to which this applies are diet, smoking, alcohol intake, and exercise. These ideas are very familiar, of course, to anyone interested in promoting BI for unhealthy behaviors, and, in specific regard to alcohol-related harm, seem to offer much promise. The recommendations were accepted by the Secretary of State for Health, Andrew Lansley, although whether they will become official government policy has yet to be seen.

The Future Forum report has been met with protests from both sides of the political spectrum. Critics on the right complain that the policy will intrude on patients’ freedom to choose how to live their lives and smacks of “the nanny state,” while characterizing the advice to be offered as “lecturing” and “preaching.” On the other hand, critics on the left argue that the recommendations are part of the government’s aim of shifting the focus of debate about health issues away from structural approaches to dealing with health inequalities in society to individual responsibility for lifestyle change. The idea that health inequalities can be addressed by personal advice on lifestyle is seen as simplistic and ideologically driven.

Whatever the merits of these opposite reactions, the point for present purposes is that the level of SBI implementation which, it has been argued in this article, would be necessary for a population-level effect to emerge—a scenario in which every time one made contact with a health professional, for any reason, one would be asked about one’s drinking and, in many cases, be advised to cut down—might not be tolerated by the general public, not to mention the health professionals asked to deliver it, and might therefore be an electoral liability to any political party supporting it.

### SBI and alcohol control measures

We reviewed above the conditions that may be necessary for a population-level effect of SBI to occur. But this assumes that SBI activity affects alcohol consumption and harms in a straightforward fashion, without interactions with other influences on drinking in society. In reality, the factors that are causally related to alcohol consumption make up a complex system of interacting forces.

One especially important set of influences is the alcohol control measures in operation in a jurisdiction at any one time. These measures refer mainly to laws and policies on taxation and their effects on the affordability of alcoholic products, the availability of alcohol, both in terms of the density of alcohol outlets and trading hours, and the marketing of alcoholic products, including advertising and promotions of various kinds. There is now a consensus among scientists in the alcohol field that these are the strongest determinants of per capita alcohol consumption [61]: under the whole population theory of alcohol problems, the level of per capita consumption determines the overall level of alcohol-related harm in a society [62,63].Policies that increase price or reduce availability and marketing are, therefore, the most effective measures for preventing and reducing alcohol-related harm [61]. But how does SBI relate to these measures?

#### Widespread SBI without alcohol control measures

Despite strong evidence for their effectiveness, alcohol control policies have seldom been implemented by governments, probably because of fears that they would be unpopular with the electorate. It has been argued that, given the reluctance of governments to enact these policies, the widespread implementation of SBI might be the only alternative for making a public health impact on alcohol-related harm [64]. However, there is also a suspicion, alluded to above, that an appeal to SBI might be used by governments to deflect attention from alcohol control measures and other societal changes necessary to reduce alcohol problems.

Beyond a direct effect on alcohol consumption and problems, however, widespread SBI might have indirect effects at community or population levels. It could, for example, encourage more health-care professionals to become involved in advocacy for effective preventive measures, including alcohol control measures [65], and perhaps influence community or even national policy agendas. Since a large proportion of the harm done by alcohol is “third-party damage” [66] (i.e., harm to others besides the drinker), widespread SBI could have benefits for families, employers, and entire communities. Probably of most importance, it might raise public awareness of alcohol-related harm and help foster the kind of “denormalization” of excessive drinking that is thought to have been crucial in reducing smoking prevalence in industrialized countries over the past few decades [67].

Another intriguing possibility is that decreases in alcohol consumption brought about in recipients of successful SBI could lead to further reductions among their family and friends by changing obligations and expectations and by modelling, which could result in a further ripple effect on drinking behavior throughout a community. This was the “snowball” mechanism proposed by Ledermann [68] to account for the fact that a decrease in the population mean of alcohol consumption did not affect the dispersion of the distribution and is a construct also central to Skog’s analysis [69] of the collectivity of drinking cultures. On an obviously speculative basis, it could be an indirect mechanism for any population-level effects widespread SBI might have.

Thus, there are reasons to believe that widespread SBI without control measures could lead indirectly to population-level reductions in alcohol-related harm, if only by increasing the public acceptability of those control measures. However, the evidence reviewed above suggests that SBI would need to be very widely disseminated indeed for this to happen, certainly above a level of dissemination that has so far occurred anywhere in the world.

#### Widespread SBI with alcohol control measures

Even if alcohol control measures were introduced by government, SBI would still have an important role to play in the battle against alcohol-related harm. This is because the two policies would have reciprocal benefits and synergistic effects.

First, it is reasonable to assume that changes in drinking behavior brought about by SBI are harder to maintain in an environment that encourages heaving drinking through the easy availability of cheap alcohol and the seductive marketing of alcoholic products. Thus, the introduction of control measures would be likely to lower the relapse rate following successful SBI. Conversely, increases in price and restrictions on availability could lead drinkers to try to cut down on their drinking, and some, especially those with some degree of alcohol dependence, might find this difficult without the help and support offered by SBI. Moreover, what was said at the beginning of this article should be reiterated: that is, the clinical benefits of SBI—the lowering of risks of harm among individual hazardous drinkers and the reductions in actual harm among harmful drinkers—exist independent of any population-level effects it might or might not have. And, however successful control measures might be in lowering per capita consumption and population harm, there would still be many individuals who would benefit from SBI.

## Conclusions

The first conclusion from this discussion is that it is impossible to be confident about an answer to the question that forms the title of this article; namely, can SBI lead to population-level reductions in alcohol-related harm? This is because of a lack of empirical evidence as to whether widespread SBI can reduce alcohol-related harm as detectable on population-level, or at least community-level, measures. Even correlational studies, such as have been conducted on the relationship between treatment for alcohol problems and population-level harm, are lacking in the SBI field. In the absence of this empirical evidence, we have to rely on estimates from modelling that suggest public health benefits would only occur if SBI were disseminated widely throughout a population. Even then, we need more assurance from long-term prospective research that reductions in consumption brought about by BI would be sustained sufficiently to enable population-level effects to occur.

There is no evidence at present that any national health care system has reached the degree of penetration of SBI necessary for population-level effects. This applies even to countries like Sweden and Finland, where the necessary conditions seem most propitious for this to happen. National mass screening programs could possibly have the desired impact but, according to the opinion of the NSC, there is a need for evidence that SBI results in reductions to alcohol-related mortality and morbidity and for the development of a simple, safe, precise, and validated screening test before standard criteria for mounting such a program can be met. There must also be a question whether the intensive dissemination of SBI that appears necessary would be politically acceptable both to health professionals and to the general public.

Widespread dissemination of SBI without the implementation of alcohol control measures might have indirect influences on alcohol consumption and harm but would be unlikely on its own to result in public health benefits. However, if and when alcohol control measures are introduced, SBI would still have an important role in the battle against alcohol-related harm.

## Competing interests

The author declares he has no competing interests.

The information in this paper was presented in part at the 8th Annual International Network on Brief Interventions for Alcohol Problems (INEBRIA) Conference, September 21–23, 2011, Boston, MA, USA.
